# Effects of 3'-sialyllactose, saliva, and colostrum on *Candida albicans* biofilms

**DOI:** 10.31744/einstein_journal/2025AO0663

**Published:** 2025-03-17

**Authors:** Juliana Barbosa Faria, Marcela Beraldo Santiago, Paula Hueb Menezes de Oliveira, Vinicius Rangel Geraldo-Martins, Ruchele Dias Nogueira

**Affiliations:** 1 Universidade de Uberaba Department of Dentistry Uberaba MG Brazil Department of Dentistry, Universidade de Uberaba, Uberaba, MG, Brazil

**Keywords:** Biofilm, Candida albicans, Colostrum, Saliva, 3'Sialyllactose

## Abstract

**Objective:**

The aim of this study was to investigate the effects of saliva, human colostrum, and 3'-sialyllactose on the initial adhesion and biofilm formation of *Candida albicans in vitro.*

**Methods:**

Colostrum and saliva samples were collected from 30 postpartum mothers and newborns, respectively. An 18h culture of *C. albicans* was treated with colostrum, 3'-sialyllactose, saliva, or a combination of colostrum and saliva at three different time points: before, during, and 24h after *C. albicans* inoculation. Biofilm assays were conducted in sterile 96-well flat-bottom microtiter plates for 24h. Biofilms were fixed, washed, stained with crystal violet, and extracted. Absorbance was measured to evaluate biofilm biomass.

**Results:**

The combined application of colostrum and saliva before and after microbial inoculation did not differ from the control biofilms (p<0.05). In contrast, the combined application of colostrum and saliva during *C. albicans* application resulted in a higher biomass than the control (p<0.05). Saliva alone decreased the biofilm biomass (p<0.05), and the application of 3'-sialyllactose reduced biofilm formation regardless of the timing of application (p<0.05).

**Conclusion:**

Saliva contributed to the proliferation of biofilms, while colostrum did not prevent initial adhesion but influenced biofilm accumulation and development. In contrast, 3'-sialyllactose significantly decreased biofilm formation at all application times. These findings underscore the importance of colostrum as a potent oral antimicrobial biofluid.

## In Brief

Faria et al. evaluated the initial adhesion and biofilm formation of *Candida albicans in vitro* in the presence of saliva, human colostrum, and 3'-sialyllactose. Colostrum applied either before or after inoculation with saliva did not differ from that of the control biofilms (p<0.05). In contrast, colostrum applied during *C. albicans* inoculation resulted in a higher biomass than the control (p<0.05). Saliva without colostrum decreased the biofilm biomass (p<0.05), and the application of 3'- sialyllactose reduced biofilm formation regardless of the timing of application (p<0.05).

## Highlights

Candidiasis can present as localized or systemic infections. Disseminated infections in newborns and adults can be life-threatening, with high mortality and morbidity rates (40-60%), and rank as the fourth most common type of nosocomial infection.

Oral candidiasis is a local condition affecting 10-15% of children in their first months of life.

Saliva and 3'-sialyllactose can disrupt the initial development of *Candida albicans* biofilm.

The effect of colostrum needs to be elucidated because it may hinder initial adhesion but promote fungal proliferation after application.

## Graphical abstract

**Figure:**
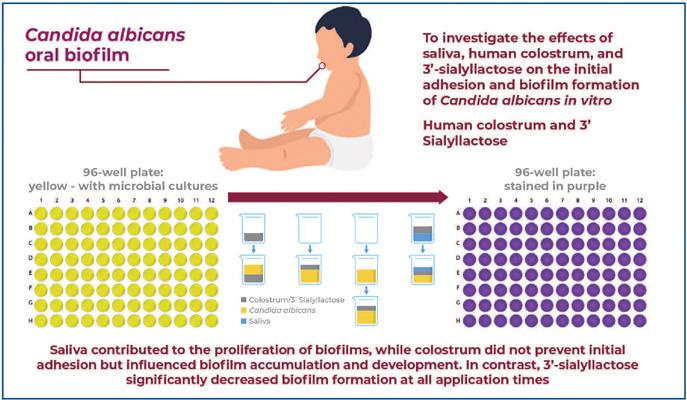

## INTRODUCTION

The oral colonization of a newborn begins at birth when the child acquires microorganisms from the birth canal, contact with other people, and the environment.^(1)^ The oral cavity is the primary entryway for various microorganisms. However, certain bacteria cannot colonize the oral cavity, even after coming into contact with the mucosa.^(2)^ Others establish residence on mucous surfaces, modify them, and facilitate the formation of complex microbial communities.^(3)^ Biofilms are the microbiological outcomes of sessile communities, characterized by cells adhering to a substrate.^(4)^ Some microbial biofilms are implicated in human illnesses,^(5)^ such as candidiasis-a group of common childhood oral diseases caused by *Candida albicans. Candida* spp. are dimorphic fungi that can exist as commensal yeast or invasive hyphae. *Candida* biofilms comprise sessile-shaped heterogeneous ecosystems that include *Candida* cells, an extracellular matrix,^(6)^ and sometimes bacteria.^(7)^

Candidiasis can present as localized or systemic infections.^(8)^ Disseminated infections in newborns and adults can be lethal, with high mortality and morbidity rates (40-60%), and are the fourth most common type of nosocomial infection.^(9)^ Oral candidiasis is a local manifestation that affects 10-15% of children in their first months of life.^(10)^ *C. albicans,* the most prevalent fungal species, is a commensal in the human oral cavity but is also an opportunistic pathogen. It can cause oral lesions when the host's immunity is compromised or hygiene is inadequate.^(11)^ Approximately 5.7% of newborns have *Candida* spp. in their oral cavity, with the prevalence increasing by 44% between 0.5 and 1.5 years of age.^(12)^ This microorganism primarily accumulates on the tongue but can spread across mucosal surfaces and saliva, organizing into biofilms that enhance its survival and expression of virulence factors.^(13)^ It has several virulence factors, including the capacity to adhere to surfaces through filament growth and produce hydrolytic enzymes that damage host cells.^(11)^

Oral colonization in the first few days and months of life in newborns is progressive and poses a challenge to the developing mucosal immune system. Newborns have a higher incidence of colonization by many microorganisms than adults or older children because of the immaturity of their immune systems.^(14)^ Saliva contains antibodies^(15)^ that control microbiota by reducing microbial adherence to the oral mucosa and teeth. Notably, saliva reduces *C. albicans* biofilm formation, as demonstrated in a study showing decreased formation in its presence.^(16)^

Current guidelines recommend exclusive breastfeeding for the first 6 months of life, followed by continued breastfeeding while introducing complementary foods. Breast milk provides nutritional and defensive components, including immunoglobulins, lactoferrin, casein, lactoperoxidase, lysozymes, leukocytes, non-adhering oligosaccharides, antiviral lipids, and antiinflammatory agents.^(17)^ These components protect infants against early infectionsand supports the developing immune system. Free oligosaccharides are natural components of milk from all placental mammals^(18)^ and are the third most abundant components after lactose and lipids^(19,20)^ Evidence suggests that human milk oligosaccharides inhibit the adherence and action of pathogens on epithelial surfaces, providing essential protection to infant health^(18)^ and contributing to immune system development.^(21)^ The predominant oligosaccharides in breast milk are 3'-sialyllactose and 6'-sialyllactose. Their concentrations vary throughout the lactation period.^(22)^ These oligosaccharides play a crucial role in the early states of infection by interfering with pathogen adherence to host cells.^(23)^ Several pathogenic microorganisms, such as *Vibrio cholera, Escherichia coli,* and *Helicobacter pylori,* recognize sialic acid-containing receptors from target cells, and sialylated oligosaccharides from human milk may function as analog receptors by inhibiting the binding of these pathogens to the cell.^(23)^ A recent study showed that 3'-sialyllactose and colostrum can decrease biofilm formation of *Streptococcus mutans in vitro,*^(24)^ a bacterium associated with dental caries.

## OBJECTIVE

To investigate the effect of 3'-sialyllactose on the formation and development of *C. albicans* biofilms *in vitro,* with or without the presence of saliva.

## METHODS

### Study design and collection

Thirty pairs of mothers and newborns were enrolled in this study after obtaining written informed consent for participation. This study was approved by the Ethics Committee of the *Universidade de Uberaba* (CAAE: 15669413.6.0000.5145; #640974). Maternal and gestational information was obtained by interviewing expectant mothers upon their arrival at the maternity hospital. The inclusion criteria were as follows: age >18 years, good general health (no systemic health issues), and no illegal drug use, tobacco use, or antibiotic or anti-inflammatory drug use. Exclusion criteria included newborns with congenital malformations, perinatal hypoxia, intracranial hemorrhage, abnormal length or weight for gestational age, or those receiving antibiotic therapy. Saliva samples were collected from newborns after birth. Colostrum was collected from the mothers 12 h after birth.

Colostrum samples were manually expressed into sterile polypropylene Falcon tubes, transported on ice to the laboratory, centrifuged at 1,300×g for 7 min to remove lipid components and stored at -80ºC until use. Unstimulated whole saliva samples were collected from the newborns before the first breastfeeding through suction using sterile Pasteur pipettes into 10 mM EDTA and kept on ice. Stock solutions of 3'-sialyllactose (10% w/v) were prepared in deionized sterile water, microfiltered using a 0.2*μ*m syringe filter (Corning, Corning, NY, USA), and stored at 4ºC

### Experimental and Control Groups

*C. albicans* biofilm formation was analyzed at different time points of colostrum, saliva, and/or 3'-sialyllactose (Sigma-Aldrich, St. Louis, MO,USA) application in microtiter plates, resulting in the following experimental groups: Group C-BE: Colostrum applied before *C. albicans* inoculation; Group C-DU: Colostrum applied during *C. albicans* inoculation; Group C-AF: Colostrum applied 24h after *C. albicans* inoculation; Group S: Saliva applied before *C. albicans* inoculation; Group C+S: Saliva and colostrum applied before *C. albicans* inoculation; Group SI-BE: 3'-Sialyllactose applied before *C. albicans* inoculation; Group SI-DU: 3'-Sialyllactose applied during *C. albicans* inoculation; Group SI-AF: 3'-Sialyllactose applied 24h after C. *albicans* inoculation; Group B24h: 24h of *C. albicans* biofilm formation; Group B48h: 48h of *C. albicans* biofilm formation.

### Yeast strain and growth conditions

*C. albicans* ATCC10231 was propagated in yeast peptone dextrose (YPD) medium. Cells were harvested, washed twice in sterile phosphate-buffered saline (PBS; 10mM phosphate buffer, 2.7mM potassium chloride, 137mM sodium chloride; pH 7.4; Sigma-Aldrich), resuspended in RPMI, and adjusted to the desired density.

### Biofilm formation

All experiments were conducted in pre-sterilized, polystyrene, flat-bottom, tissue culture-treated 96-well microtiter plates (Thermo Fisher Scientific, Waltham, MA, USA). Standardized cell suspensions (lOOmL portions of suspensions containing 1.0×10^6^ cells/mL in RPMI, adjusted to an optical density [OD] of 1.0 at 595nm) were seeded into selected wells of the microtiter plates and treated according to the described groups. Briefly, 5*μ*L of saliva, colostrum, and 3'-sialyllactose were applied to the wells. The plates were incubated aerobically for 24h at 37ºC. After the initial incubation, the medium was aspirated, and non-adherent cells were removed by washing three times with sterile PBS.

The attached yeast cells were fixed with 1mL of 10% formaldehyde solution and left overnight at room temperature. The microplates were emptied and airdried. Each well was stained for 15 min with 0.2mL of 0.1% (w/w) crystal violet and incubated at room temperature for 1h. The crystal violet solution that did not adhere to the biofilm was removed by washing twice with distilled water. The microplates were air-dried, and the dye bound to the adherent cells was removed with 0.2mL of 30% (v/v) glacial acetic acid per well. The OD of the resulting solutions was measured at 490nm using a microplate reader (Molecular Devices, Inc., Sunnyvale, CA, USA).

### Statistical analysis

Experiments for colostrum and 3'-sialyllactose were repeated three and ten times, respectively. The results were evaluated by measuring the OD after crystal violet staining. The mean OD values were used to assess biomass formation and development in groups treated with colostrum samples before, during, and after biofilm formation, and were compared with 24-and 48h biofilm controls. Comparisons of biomass quantity between groups were analyzed by analysis of variance (ANOVA), followed by post hoc analysis using the Bonferroni test. The frequencies of enhanced or diminished biofilms were compared among the groups using the χ^2^ and Fisher's exact tests. Statistical significance was set at p<0.05.

## RESULTS

**Figure 1.**
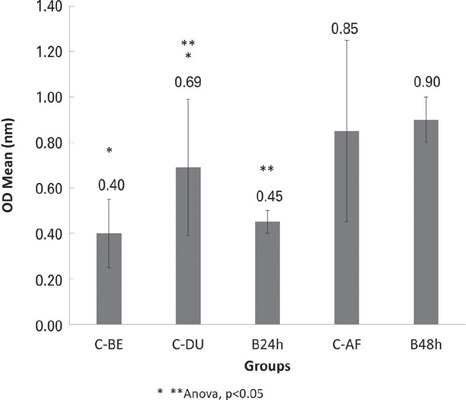
Optical density (0D) of *Candida albicans* biofilm biomass in the groups in which colostrum was applied before (C-BE), during (C-DU), and after microbial inoculation (C-AF), compared with the 24h (B24h) and 48h (B48h) *C. albicans* biofilms (controls)

The biofilms formed after 24h had an average OD of 0.45±0.05, which was significantly smaller than the biofilms formed after 48h (0.90±0.10) (p<0.05). There was no significant difference in the mean OD of the yeast biomass between the C-BE and Control (B24) Groups (Figure 1; p>0.05). In contrast, the simultaneous incubation of colostrum and yeast inoculum (Group C-DU) significantly increased biofilm development and accumulation after 24h compared to the B24 control (Figure 1; p<0.05). There were significant differences between the ODs of the biofilms in the groups that received colostrum before and during inoculation (CBE *versus* C-DU) (Figure 1; p<0.05). The application of colostrum 48h after microbial inoculation (Group C-AF) did not affect biomass formation, as no significant differences were observed between the ODs of the C-AF and B48h Groups (Figure 1; p>0.05).

**Figure 2.**
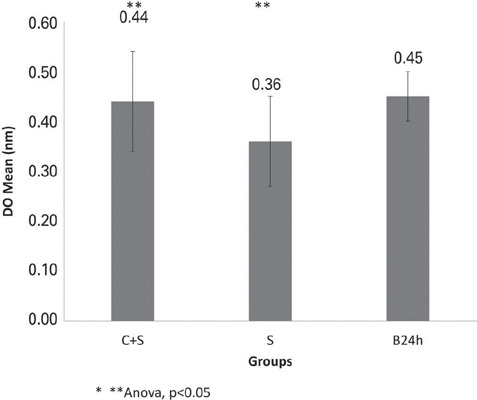
Optical density (OD) of *Candida albicans* biofilm biomass in the groups in which saliva (CA-S) and a combination of colostrum and saliva (C + S) were applied before microbial inoculation, compared with the 24h *C. albicans* biofilm (B24h; control)

The mean OD of the group treated with saliva (S) before inoculation was significantly lower than that of the B24h Group (Figure 2; ANOVA, p<0.05). However, the presence of saliva in conjunction with colostrum (C+S) did not reduce biofilm formation compared to that in the B24h Group (Figure 2; ANOVA, p>0.05). A statistically significant difference was observed between the ODs of the S and C+S Groups (Figure 2; p<0.05). Comparisons between the application of colostrum and saliva showed no significant differences in biofilm OD when applied before inoculation (C-BE *versus* S; Figure 1 and 2; p>0.05). However, the application of colostrum alone before inoculation (Group C-BE) reduced OD more effectively than the combined application of saliva and colostrum (Group C+S; Figure 1 and 2; ANOVA, p<0.05).

**Figure 3.**
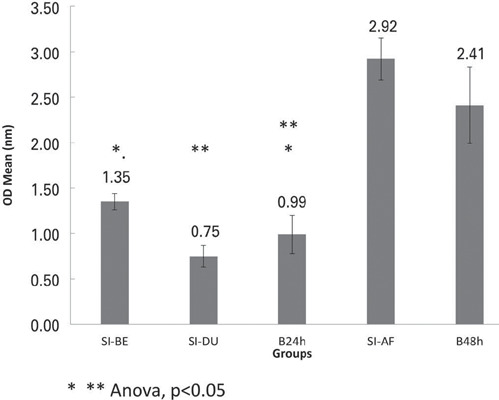
Optical density (OD) of *Candida albicans* biofilm biomass in the groups in which 3'-sialyllactose was applied before (SI-BE), during (SI-DU), and after (SI-AF) microbial inoculation, compared with the 24h (B24h) and 48h (B48h) *C. albicans* biofilms (controls)

The application of 3'-sialyllactose during (Group SI-DU) and before (Group SI-BE) *C. albicans* inoculation significantly reduced biofilm OD after 24h compared to the Control Group (Figure 3; ANOVA, p<0.05). However, no significant differences were observed when 3'-sialyllactose was applied 24h after *C. albicans* inoculation (Group SI-AF; ANOVA, p>0.05).

The frequencies of samples that showed an increase or decrease in *C. albicans* biofilm formation are shown in table 1. Most colostrum samples, when applied before or after biofilm formation (Groups C-BE and C-AF) or in combination with saliva (Group C+S), reduced biofilm formation (Table 1). However, when colostrum was applied during microbial inoculation (Group C-DU), biofilm formation increased in 87% of the tested samples. The frequency of samples exhibiting increased biofilm formation in the C-DU Group was significantly higher than that in the other groups (p<0,05). Furthermore, no significant differences in the frequency of samples that reduced biofilm formation were observed among the C-BE, C-AF, and C+S Groups (Table 1; p>0.05). In contrast, Group S, which involved the application of saliva before microbial inoculation, exhibited the highest frequency of samples (93%) that reduced biofilm formation and was statistically different from the other groups (Table 1; p<0.05).

**Table 1:** Optical density of *Candida albicans* biofilm biomass in the groups in which colostrum (C-AF), saliva (S), and a combination of colostrum and saliva (C+A) were applied before, during, and after microbial inoculation, compared with the 24h *C. albicans biofilm* (B24h; control)

Groups (n=30)	Incresead n (%)	Decreased n (%)
C-BE	10 (33)^1^,^5^	20 (67)^1^,^5^
C-DU	26 (87)^1^,^2^,^3^,^4^	4 (13)^1^,^2^,^3^,^4^
C-AF	11 (37)^2^,^6^	19 (63)^2^,^6^
C+S	11 (37)^3^,^7^	19 (63)^3^,^7^
S	2 (7)^4^,^5^,^6^,^7^	28 (93)^4^,^5^,^6^,^7^

1, 2, 3,4, 5, 6 and 7 are the quartets performed in the statistical analyses, p<0.05.

## DISCUSSION

Colonization by *Candida* spp. has been observed in 49.1% of infants in their first month of life. *C. albicans* is the most prevalent species in the oral cavity. Adhesion is a crucial step in *Candida* pathogenicity and biofilm formation. Blocking this process may prevent fungal colonization on the oral epithelial surface. The formation of *C. albicans* clusters in biofilms is one of the main virulence mechanisms of yeasts, making them less susceptible to antimicrobial action.^(25)^ Fungal adherence to host cells is mediated by adhesins, whose expression is influenced by environmental and host-related factors. These adhesins are essential for fungal survival on host surfaces, attachment to epithelial cells, and internalization.^(26)^

Here, we aimed to analyze the development of *C. albicans* biofilm on polystyrene plates in the presence of colostrum or 3'-sialyllactose at three time points: before microbial inoculation, during inoculation, and 24h after inoculation. Additionally, biofilm formation was analyzed in the presence of saliva alone and in combination with colostrum before microbial inoculation.

A previous study on the antibacterial properties of milk and colostrum revealed their potential for treating and preventing microbial diseases. Specific antimicrobial milk proteins interact with various molecules and are associated with infection control in different environments. The results showed that the application of colostrum before or during fungal inoculation did not significantly alter the average OD compared to that of the controls. However, frequency analysis revealed that more than 63% of the samples showed a reduction in biofilm formation. Protective factors such as immunoglobulin A (IgA)^(27)^ and lactoferrin, are known to inhibit *C. albicans* proliferation in animal models.^(28)^ They are important protein agents with antimicrobial effects and offer protection against fungi, especially *Candida* spp.

Although 93% of the salivary samples reduced biofilm formation, the effects of saliva on *C. albicans* biofilms reported in literature remain variable. Xerostomia and other saliva production disorders, often associated with decreased IgA levels, can lead to increased *Candida* colonization.^(29)^ Previous studies have pointed out that many salivary components may inhibit fungal populations.^(30)^ Molecules such as lysozymes, lactoferrins, histatins, peroxidases, calprotectins, and salivary IgA prevent fungal adherence to prosthetic surfaces. However, other salivary components, including mucins, statherins, and proline-rich proteins, facilitate fungal adhesion and colonization.^(30)^ This dual role of saliva may explain the growth of biofilms observed in colostrum applications during fungal inoculation.

The identification of specific breast milk components, particularly oligosaccharides, has garnered substantial interest. Oligosaccharides have a beneficial effects on the composition of infant microbiota.^(31)^ Infants fed cow milk or formulae not supplemented with oligosaccharides have microbiomes with fewer bifidobacteria, which can negatively affect immune development.^(32)^ Evidence suggests that oligosaccharides act as soluble ligand analogs, blocking the binding of pathogens to epithelial cells.^(33,34)^ Our findings demonstrated that 3'-sialyllactose reduced the biofilm formation when applied before or during *C. albicans* inoculation. This indicates that this oligosaccharide may interfere with early microbial adherence, which aligns with our previous study showing that 3'-sialyllactose inhibits *Streptococcus mutans* adherence *in vitro.*^(24)^ Additionally, 3'-sialylactose has been shown to reduce the adhesion of *Clostridium difficile* to human colon cells *in vitro*^(35)^ and inhibit the binding of *Vibrio cholera, Escherichia coli,* and *Helicobacter pylori* to intestinal cells.^(23)^

## CONCLUSION

Our fndings suggest that while saliva promotes biofilm proliferation, colostrum does not prevent initial adhesion but influences biofilm accumulation. Interestingly, 3'-sialyllactose consistently reduced biofilm formation, regardless of the timing of its application. These results highlight the antimicrobial potential of biofluids, emphasizing their role in reducing fungal colonization and offering potential therapeutic insights. Furthermore, these findings underscore the need for further research to optimize their application in clinical or preventive contexts.
